# Exploring Individual Variability in Drug-Induced Liver Injury (DILI) Responses through Metabolomic Analysis

**DOI:** 10.3390/ijms25053003

**Published:** 2024-03-05

**Authors:** Marta Moreno-Torres, Guillermo Quintás, Teresa Martínez-Sena, Ramiro Jover, José V. Castell

**Affiliations:** 1 Unidad Mixta de Hepatología Experimental, Instituto de Investigación Sanitaria del Hospital La Fe (IIS La Fe), 46026 Valencia, Spain; marta.moreno.torres@uv.es (M.M.-T.); ramiro.jover@uv.es (R.J.); 2 Departamento de Bioquímica y Biología Molecular, Facultad de Medicina y Odontología, Universidad de Valencia, 46010 Valencia, Spain; 3 Centro de Investigación Biomédica En Red de Enfermedades Hepáticas y Digestivas CIBEREHD, Instituto de Salud Carlos III, 28029 Madrid, Spain; 4 Metabolomics and Bioanalysis, Leitat Technological Center (LEITAT,) 46026 Valencia, Spain

**Keywords:** DILI, idiosyncratic DILI, metabolomics, hepatocellular damage, cholestasis, hepatotoxicity, biomarkers, updated RUCAM

## Abstract

Drug-induced liver injury (DILI) is a serious adverse hepatic event presenting diagnostic and prognostic challenges. The clinical categorization of DILI into hepatocellular, cholestatic, or mixed phenotype is based on serum alanine aminotransferase (ALT) and alkaline phosphatase (ALP) values; however, this classification may not capture the full spectrum of DILI subtypes. With this aim, we explored the utility of assessing changes in the plasma metabolomic profiles of 79 DILI patients assessed by the RUCAM (Roussel Uclaf Causality Assessment Method) score to better characterize this condition and compare results obtained with the standard clinical characterization. Through the identification of various metabolites in the plasma (including free and conjugated bile acids and glycerophospholipids), and the integration of this information into predictive models, we were able to evaluate the extent of the hepatocellular or cholestatic phenotype and to assign a numeric value with the contribution of each specific DILI sub-phenotype into the patient’s general condition. Additionally, our results showed that metabolomic analysis enabled the monitoring of DILI variability responses to the same drug, the transitions between sub-phenotypes during disease progression, and identified a spectrum of residual DILI metabolic features, which can be overlooked using standard clinical diagnosis during patient follow-up.

## 1. Introduction

Drug-induced liver injury (DILI) is an unexpected toxic effect of bioactive compounds, not linked to their intrinsic pharmacological properties of the drug. This event can occur during the initial clinical phases of drug development as well as during the widespread clinical use of a drug, including self-consumption of over-the-counter drugs containing bioactive compounds. Hepatotoxicity is among the major reasons for drug development discontinuation and withdrawal from the market, representing a major safety and economic concern for the pharmaceutical industry and health systems. Although DILI is in most cases self-remitting when the administration of the responsible drug is stopped [1,2,3], in rare cases, it may lead to a potentially serious adverse reaction that can result in a spectrum of liver problems, ranging from mild abnormalities in liver function tests to severe hepatitis, liver failure [4], or even death [5], making it particularly challenging to understand and manage [6]. DILI stands as the predominant trigger for acute liver failure (ALF) in Western regions, boasting a case fatality rate ranging from 10% to 50% [6]. Annually in the United States, there are around 2000 instances of acute liver failure, with drugs contributing to over half of these cases (39% attributed to acetaminophen and 13% to idiosyncratic reactions from other medications). Among patients hospitalized with jaundice, drugs account for 2–5% of cases, and approximately 10% of all acute hepatitis cases are linked to drug use [7].

In addition, the incidence of DILI events is rising in parallel to the introduction of new drugs, increasing life expectancy and poly-medication in elderly people, and the widespread use of self-prescribed complementary dietetic or herbal products.

Within hepatotoxins causing DILI, the most easily identifiable ones are those having dose-dependent effects in all individuals exposed. On the contrary, idiosyncratic DILI (iDILI) stands out as an often-unpredictable phenomenon occurring in certain individuals. iDILI refers to liver injury caused by a drug or medication in a manner that is not clearly dose-dependent, sometimes with superimposed immunological hypersensitivity features that occur only in certain patients and also strongly linked to a patient’s characteristics (genetic, metabolic, etc.) [8,9]. However, the mechanisms underlying iDILI remain poorly understood.

RUCAM, a widely recognized diagnostic scale, effectively evaluates cases of DILI by utilizing well-documented clinical characteristics [10,11]. DILI clinical diagnosis involves anamnesis and differential diagnosis, as patients often exhibit nonspecific symptoms like fatigue, jaundice, abdominal pain, and nausea, and increased liver tests which can resemble other liver disorders or common illnesses [12]. The clinical DILI outcome typically ranges from hepatocellular damage to cholestasis phenotype, which is biochemically identified using the “R score”. This score is calculated as a ratio: (patient’s ALT (alanine aminotransferase, ALT)/upper limit of ALT normality)/(patient’s ALP (alkaline phosphatase, ALP)/upper limit of ALP normality). It aids in classifying the DILI episode as cholestatic (R score < 2), mixed (2 < R score < 5), or hepatocellular (R score > 5) [10,11]. Hepatocellular injury primarily affects hepatocytes, which are the main functional cells of the liver, while cholestatic injury may also involve affectation of cholangiocytes and the bile ducts, disrupting the bile flow. However, DILI can be a complex event, and its classification based exclusively on these two liver enzymes may not capture the interindividual variability and the full array of DILI subtypes existing between the pure cholestatic and the pure hepatocellular injury patterns [9,12]. Thus, significant progress is required to enhance the precision of DILI classification phenotypes and effectively translate biochemical data into decision making within clinical practice.

Metabolomics is a valuable approach for characterizing the metabolic pathways and networks of biological systems [13]. Our approach involves scrutinizing pertinent metabolic alterations occurring in the liver during a DILI event, which manifest in the patient’s sera as well.

**Figure 1:**
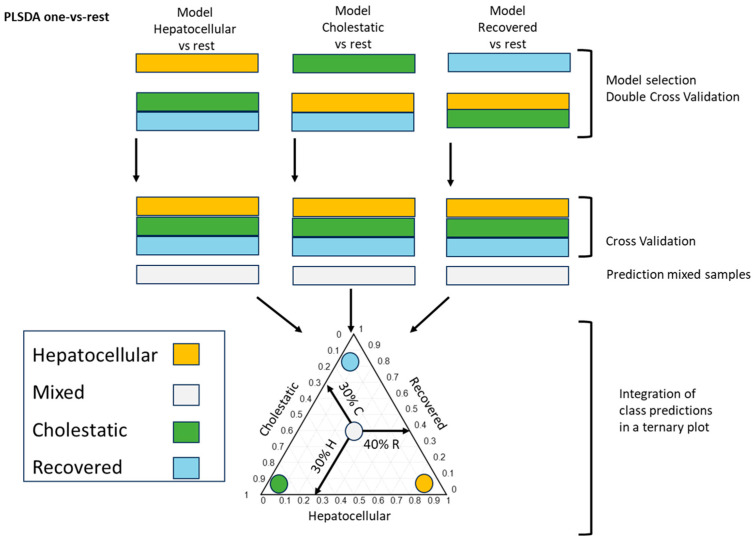
Schematic workflow of data analysis and modeling strategy. Ternary plot employed to characterize the distinct DILI phenotypes, including cholestatic, hepatocellular, mixed, and those observed in recovered patients. PLS-DA models were performed to compare hepatocellular DILI versus a group consisting of cholestatic DILI and recovered patients, cholestatic DILI versus a group comprising hepatocellular DILI and recovered patients and recovered patients versus a group encompassing hepatocellular and cholestatic DILI patients. The PLS predicted values using the three models from metabolomics data were graphically represented using ternary plots. The position of the sample in the ternary diagram should be interpreted as follows, considering 0 and 1 in the axis scale as 0% and 100% of each corresponding phenotype. In the mixed phenotype sample (white) selected as example, the arrows indicate the contribution of each DILI phenotype to the patient’s current status, a 30% of cholestatic DILI phenotype (C), 40% of recovered phenotype (R), and 30% of hepatocellular DILI phenotype (H). Each color indicates the phenotype of the samples according to the R score-based clinical classification: green: cholestatic; orange: hepatocellular; white: mixed; blue: recovered patient. Figure adapted from Quintás et al. [16].

Preceding metabolomics studies have supported its utility in evaluating hepatotoxicity [14,15]. A previous study on DILI identified metabolomic alterations in the patient’s plasma associated with the type and severity of the DILI event, enabling an accurate classification and the follow up of the patient’s clinical progression. The study identified conjugated bile acids and glycerophospholipids as the most significant classes of metabolites discriminating the different DILI phenotypes [16]. Multivariate models enabled the discrimination among the various DILI phenotypes and the recovered status according to the metabolome analysis. The analysis included the development of three Partial Least Squares Discriminant Analysis (PLS–DA) models, discriminating hepatocellular vs. cholestatic and recovered patients, cholestatic vs. hepatocellular and recovered patients, and recovered vs. hepatocellular and cholestatic DILI patients. Model development included a first step of model optimization using double-cross validation, before its application to new samples. To allow a visual identification of the severity and extent of the different DILI phenotypes, the PLS predicted values using the three models were graphically represented within ternary plots. Thus, the position in the ternary diagram summarizes the patient’s metabolomic status at a specific time point, which can be interpreted as a summary of the contribution of each DILI phenotype to the patient’s current status, offering insight into differences among mixed-type DILI cases (see Figure 1).

This study explored the utility of assessing changes in the plasma metabolomic profiles of DILI patients to characterize this condition better and numerically, and compare results obtained with the standard clinical characterization. For drugs inducing iDILI, examining the impact of DILI in the metabolome may help to evidence the individual’s response to drugs (variability in response), where factors such as genetics, environment, and lifestyle are likely involved in developing individual metabolic DILI patterns, thus providing better understanding of the different individuals responses to bioactive compounds. Additionally, this study assessed the different metabolomic profiles among DILI patients exposed to the same drug, evidencing the occurrence of variability in the observed DILI phenotypes. Furthermore, the results showed that metabolomic analysis enabled the monitoring of transitions between sub-phenotypes during disease progression, and identified a spectrum of residual DILI metabolic features which can be overlooked using standard clinical diagnosis during patient follow-up. Altogether, these findings underscore the value of metabolomics for better characterization and monitoring of DILI events over time.

## 2. Results

### 2.1. Metabolomic Analysis of Plasma during DILI Monitoring

This study involved the analysis of metabolomic profiles of 278 plasma samples collected from a cohort of 79 DILI patients exposed to 31 different drugs, using PLS-DA to discriminate between cholestatic, hepatocellular, and recovered DILI patients using a strategy based on the applications of three one-vs-rest independent PLS-DA. The results from the PLS–DA models were integrated into a ternary diagram to display the disease phenotype, the severity of the liver damage, and its progression, as described elsewhere [14]. For a few drugs (i.e., epistane, oxaliplatin, azathioprine, acetaminophen, amoxicillin-clavulanic acid, and methotrexate), plasma samples were collected sequentially after the DILI onset. According to clinicians’ expertise and the R score, 34 samples collected along the episode were classified as hepatocellular, 79 as cholestatic, and 54 as mixed-type DILI. Finally, 111 samples collected had the features of minimally injured or recovered patients (see Table S1). This table includes information regarding the coded patient ID, days from DILI onset, time-points of collected samples, R-Score, drug-inducing DILI, collection time, DILI type, ALT, ALP, total bilirubin, and the predicted percentage of each sub-phenotype, (cholestasis, hepatocellular or recovered condition), calculated from the PLS-DA predictive models.

**Figure 2:**
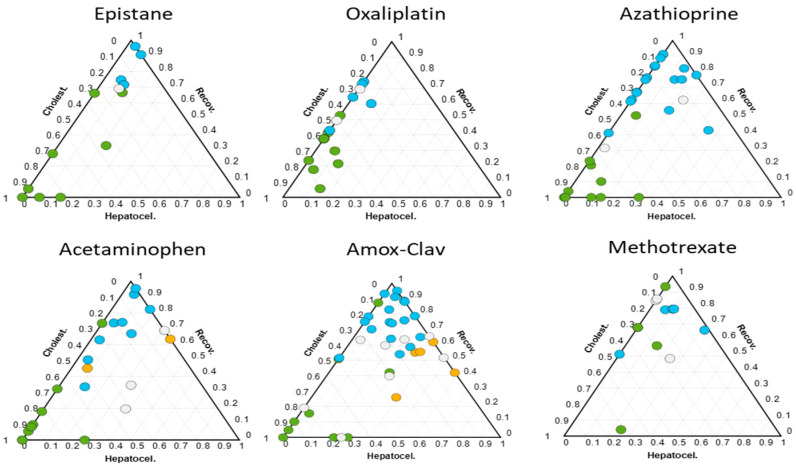
Ternary plots obtained from the analysis of samples collected from DILI patients. Each plot represents patients for whom a specific drug was identified as the causative agent responsible for the DILI event. Dot color represents the R score-based clinical classification; green: cholestatic; orange: hepatocellular; white: mixed; blue: recovered patient.

Figure 2 shows ternary plots built using the predicted values from the metabolomic analysis of samples and displays the classification scores of the DILI events among patients exposed to each drug. The results depicted indicate that certain drugs exhibited a consistent DILI metabolomic phenotype in all individuals and at all stages of the disease. Epistane, an anabolic steroid misused by bodybuilders to enhance muscle mass, is a cholestatic agent that causes elevated levels of cholic acid conjugates by upregulating bile acid synthesis (CYP8B1) and cross-talking with nuclear receptors in human hepatocytes [17]. Oxaliplatin, a platinum containing alkylating agent currently used for the treatment of advanced colorectal cancer and other neoplastic diseases [18], is associated with severe hepatic sinusoidal obstruction [18,19]. Azathioprine is a medication generally used in the management and treatment of active rheumatoid arthritis (RA) and the prevention of kidney transplant rejection [20]. Azathioprine-induced cholestatic hepatitis has been reported in various settings including renal and cardiac transplantation [21]. The results shown in Figure 2 demonstrate that these drugs induced in all cases a cholestatic phenotype that persisted during disease course until recovery.

Acetaminophen is a drug whose liver injury mechanism involves bioactivation to a quinone imine, redox cycling, subsequent depletion of glutathione (GSH), covalent binding to proteins and ultimately hepatocellular damage and necrosis [22]. Although acetaminophen toxicity is often associated with pure hepatocellular damage, the results depicted in Figure 2 showed that, in this study, it led to significantly different DILI metabolic phenotypes across patients ranging from pure hepatocellular to mixed and cholestatic. These results suggest that the induced hepatocellular damage affected liver bile function as well resulting in a greater or lesser extent of cholestasis. Amoxicillin-clavulanic typically induces cholestasis of severe phenotype [23,24,25]. However, the analysis of the plasma samples collected from DILI patients taking this drug showed a wide variability in the metabolic phenotype with individuals displaying from a cholestatic to a hepatocellular phenotype (see Figure 2). Methotrexate is an antineoplastic and immunosuppressive agent widely used in the therapy of leukemia, lymphoma and several solid tumors that can lead to acute elevations in serum liver enzymes and, in some cases, development of chronic liver injury, progressive fibrosis, cirrhosis and portal hypertension [26]. Although the damage caused by the drug in the patients included in this study was moderate, some individuals displayed a clear cholestatic phenotype.

**Figure 3:**
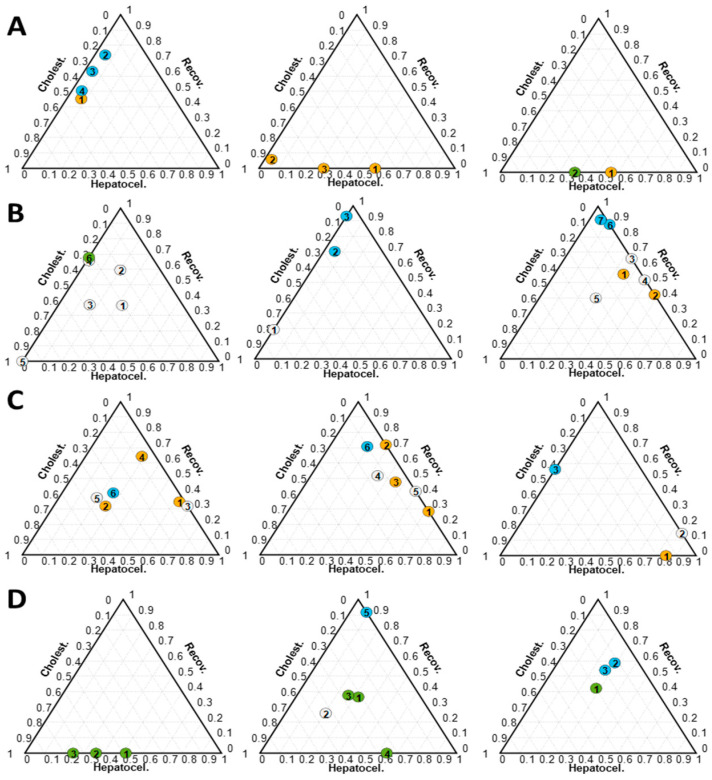
Ternary plots obtained from the analysis of samples collected from DILI patients. Each plot represents results from the analysis of samples collected from a single patient. The number inside each circle indicates the order of collected samples and is meant to identify the patients’ metabolome over time after the onset of the DILI event. Dot color represents the R score-based clinical classification; green: cholestatic; orange: hepatocellular; white: mixed; blue: recovered patient. (**A**) Patients 62, 24, and 59 (from left to right) classified as hepatocellular (R score > 5) with a cholestatic (62) and mixed (24 and 59) metabolomic profile; (**B**) patients 30, 102, and 34 (from left to right) classified as mixed (2 < R score < 5) with a cholestatic metabolic profile; (**C**) patients 17, 114, and 137 (from left to right) classified as mixed (2 < R score < 5) with a hepatocellular metabolic profile; (**D**) patients 48, 136, and 80 (from left to right) classified as cholestatic (R score < 2) with a metabolomic mixed phenotype (see also Table S1).

These results indicated substantial variations in patients’ responses when exposed to the same causative agent, revealing considerable variability among individuals. Figure 3 shows the predicted values for samples collected from 12 patients clinically diagnosed as cholestatic, hepatocellular or mixed DILI based on the R score. The findings showed that patients who were clinically categorized within a particular DILI sub-phenotype nevertheless exhibited significant differences in their metabolomics profiles (see Table S1).

**Figure 4:**
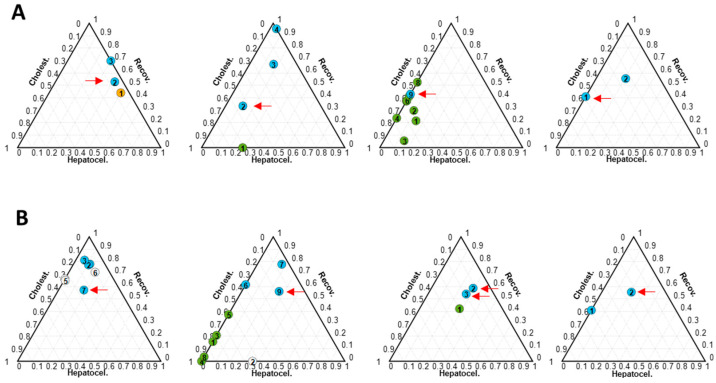
Ternary plots obtained from the analysis of samples collected from DILI patients clinically classified as recovered. Each plot represents results from the analysis of samples collected from a single patient. All patients represented were classified as recovered based on biochemical parameters at the times indicated by the red arrows. (**A**) Patients 14, 51, 89, and 108 (from left to right), and (**B**) patients 49, 78, 80, and 108 (from left to right). Dot color represents the clinical classification at each point: green: cholestatic DILI; orange: hepatocellular DILI; white: mixed DILI; blue: recovered patient. The number inside each circle indicates the order of collected samples and is meant to identify the patients’ metabolome over time after the onset of the DILI event. Each ternary plot represents the results of a specific individual and the number within each dot indicates the timing of the samples.

Furthermore, clinical classification identifies recovered patients as those with ALT < 56 unit/L, ALP < 147 unit/L and bilirubin (BIL) < 1.2 mg/dL in the absence of any other clinical or analytical sign of disease [16]. However, in some cases, the metabolomic profiles of patients clinically classified as recovered still matched moderate cholestatic or hepatocellular phenotypes. The observed mismatch between clinical classifications based on the R score and the metabolomic based results suggest an asynchronous evolution profiles between the ALT, ALP, and BIL values and the metabolic profiles towards normalization during DILI recovery and underscores the value of metabolomics data in uncovering the complex metabolic changes underlying a DILI event. Metabolomics offers a more comprehensive description of alterations within plasma samples compared to the insights provided solely by the R score. For example, patient 14-t2, despite being classified as recovered according to biochemical parameters (ALT = 33, ALP = 103, BIL= 1.63 mg/dL) displayed a hepatocellular metabolic phenotype (41% H, Table S1). Additionally, while the patient samples 51-t2 (ALT = 32, ALP = 119, BIL = 4.02), 89-t9 (ALT = 30, ALP = 124, BIL = 0.98), and 108-t1 (ALT = 43, ALP = 145, BIL = 0.24) were classified as recovered, the metabolomics results indicated the occurrence of cholestatic profiles (54% C, 57% C and 59% C, respectively) (Figure 4A and Table S1). For the patient samples 49-t7 (25% C and 18% H), 78-t9 (18% C and 26% H), 80-t2 (12% C and 29% H), 80-t3 (19% C and 27% H), and 108-t2 (24% C and 21% H), also clinically classified as recovered, a significant contribution of the cholestasis and/or hepatocellular sub-phenotypes was still remarkable (Figure 4B and Table S1).

These results suggest a potential sub-classification of the recovered cohort into two groups: individuals who have fully recovered and those that still exhibit concern due to lingering DILI-related features, warranting continued monitoring.

### 2.2. Analysis of Progression Patterns after DILI Diagnosis

**Figure 5:**
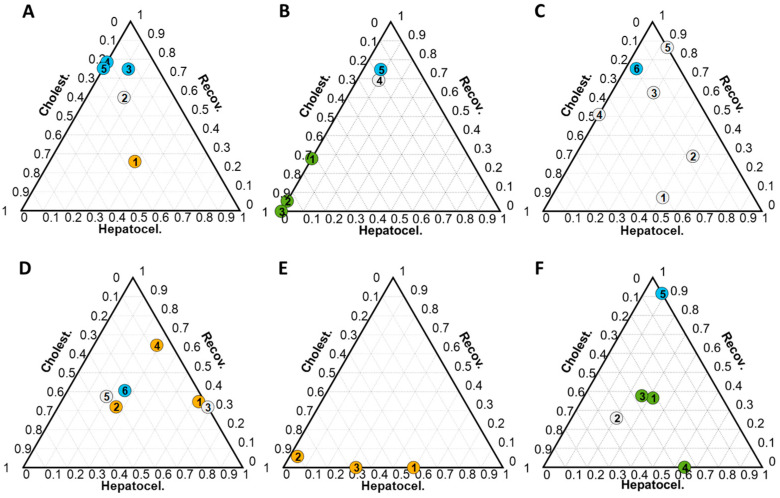
Ternary plots obtained from the analysis of samples collected from DILI patients during monitoring. Each plot represents results from the analysis of samples collected from a single patient and the number within each dot indicates the timing of the samples. Dot color represents the clinical classification at each point: green, cholestatic DILI; orange: hepatocellular DILI; white: mixed DILI; blue: recovered patient. Ternary plots of (**A**–**C**) patients 87, 94, and 36 (from left to right) and (**D**–**F**) patient samples 17, 24, and 136 (from left to right) with a DILI progression within the same sub-phenotype towards recovery or not.

Ternary plots summarizing the outcomes from the metabolomic analysis were employed to monitor the evolution of the metabolome of patients over time following DILI diagnosis. Most of the patients included in this study presented an evolution from hepatocellular (Figure 5A), cholestatic (Figure 5B), or mixed-type phenotypes (Figure 5C) towards recovery.

Nonetheless, the analysis revealed a shift in the metabolomic phenotypes during the recovery phase and some patients transitioned from a hepatocellular to cholestatic injury pattern or vice versa, indicating changes in the type of mechanism of liver damage during the disease. That was the case of patient 17, who was initially diagnosed as hepatocellular (t1 = 3% C and 63% H), but this evolved by changing the phenotype at different time points (t) such as t2 (42% C and 26% H), t5 (43% C and 19% H), and t6 (33% C and 26% H) towards a predominant cholestatic phenotype (Figure 5D and Table S1). A similar example occurred in the case of patient 24, who was clinically diagnosed as pure hepatocellular, although it presented a mixed DILI phenotype according to metabolomics and by shifting its pattern towards a cholestatic phenotype (t1 = 41% C and 59% H, t2 = 91% C and 4% H, and t3 = 67% C and 33% H) (Figure 5E and Table S1).

An opposite trend is shown for patient 136, whose time point samples t1, t2, and t3 displayed a small percentage (30% approximately) of hepatocellular DILI phenotype which increased up to 65% at t4 (Figure 5F, Table S1).

Thus, longitudinal analysis of DILI metabolic phenotypes disclosed that, while in some cases there is a continuous trend towards recovery upon drug discontinuation, in other cases, the phenotype may evolve and change from hepatocellular to cholestatic or mixed or vice versa.

## 3. Discussion

The patients’ serum ALT and ALP values are used for the calculation of the R score, a widely used parameter for the classification of DILI phenotypes as hepatocellular, cholestatic, or mixed type. However, this classification, which is essentially based on the relative ratio of hepatocyte’s transaminase ALT over hepatocyte-cholangiocyte’s phosphatase alkaline ALP, lacks precision in appreciating subtle differences within intermediate mixed types of DILI and the magnitude, extent, and the DILI progression. Some limitations arise particularly in cases involving specific toxicity mechanisms [27]. For instance, hepatocellular DILI resulting from early-stage inhibition of the mitochondrial respiratory chain may not be reflected in elevated ALT or ALP values [28]. Moreover, ALT and AST are not specific to the liver but also to muscle and cardiac damage [29,30]. Moreover, ALT, ALP, and AST are not specific to the etiology of liver damage, and baseline alterations may be present in individuals with prior liver diseases [31]. Mixed-type DILI introduces uncertainty, as liver enzyme levels may not reliably correlate with histological patterns [32]. Elevated ALT levels during treatment with potentially hepatotoxic drugs may normalize despite ongoing cellular dysfunction [31]. Finally, the extended half-life of transaminases poses challenges for dynamic monitoring, and the nature of toxic liver injury can evolve over the course of the illness, with specific drugs not consistently associated with distinct damage patterns [33]. Given the liver’s crucial role in regulating the body’s metabolic processes that can be disrupted by the interfering effects of a drug, it was reasonable to assume that the effects of a hepatotoxic drug causing DILI would be reflected in changes within the cell’s metabolome and exo-metabolome. Therefore, studying the impact on the metabolome provides a more comprehensive insight into DILI outcomes, revealing the intricate individual responses to drugs, and uncovering DILI variability which could be missed by more conventional scores. For that purpose, a cohort study involving 79 DILI patients, assessed by the updated RUCAM, treated with 31 different drugs was recruited for the analysis of the variation of their plasma metabolome during the DILI event after diagnosis [16]. Several metabolites including free and conjugated bile acids and glycerophospholipids were determinant for accurate classification of the DILI sub-phenotypes provided by an ensemble of PLS–DA models. In the present work, we explored the feasibility of metabolomics analysis to evidence phenotype variability among patients experiencing a DILI event caused by the same drug. This approach allowed the identification of a potential sub-classification of recovered patients into two groups, those who have achieved complete recovery and those who continue to display concerns associated with persistent DILI features; and it permitted us to analyze the different progression patterns of each patient after DILI diagnosis.

Among the 31 different drugs evaluated, we identified two different drug categories. The first category, including epistane, oxaliplatin, and azathioprine, consistently led to the same phenotype in all patients who were exposed. The second category comprised drugs like acetaminophen, amoxicillin-clavulanic, and methotrexate, which exhibited variable and patient-related metabolome features. Despite being exposed to the same drug, patients displayed different DILI sub-phenotypes, sometimes changing along the DILI event, within this category of drugs.

The cholestatic effect of epistane has been attributed to an induced bile acid synthesis that favored bile acid accumulation in hepatocytes at least in part by the androgen receptor activator. Regarding epistane, it was speculated that the significant phenotypic diversity observed in human bile acid synthesis enzymes and transporters offers a potential explanation of this idiosyncratic occurrence [17].

Regarding oxaliplatin, it is a drug potentially causing adverse episodes of hepatotoxicity with cholestasis being a documented event. This has been associated with mild-to-moderate histological changes in the liver by sinusoidal dilation, congestion, and sinusoidal obstruction syndrome (SOS). This obstruction can result in congestion and damage to liver cells, and it can compromise the normal flow of bile, causing cholestasis [34,35]. According to the metabolomic analysis, cholestasis was the predominant and consistent phenotype detected in all studied cases from our cohort.

Azathioprine hepatic injury typically resolves promptly upon discontinuation of the medication. It is characterized for its tendency to temporarily elevate liver enzyme levels and potentially trigger cholestasis with subsequent development of hepatic ductopenia [21,36]. Indeed, the findings in our study were consistent in azathioprine inducing cholestatic phenotype in all patients from our cohort.

Although acetaminophen has generally been used as an example of intrinsic DILI, where liver toxicity (necrosis) is induced in a predictable and dose-related necrosis, it is also displaying variable DILI responses. While acetaminophen toxicity by overdose is primarily associated with hepatocellular injury, variable responses of DILI were observed, including a cholestatic sub-phenotype [37,38]. This variability in responses is well evidenced in the metabolomics analysis of individuals of our cohort that displayed a range between hepatocellular and cholestatic phenotypes.

Regarding amoxicillin-clavulanic (AC), class I and II HLA (human leukocyte antigens) genotypes have been shown to affect susceptibility to amoxicillin-clavulanic-DILI, indicating the importance of the adaptive immune response in pathogenesis; however, they still have limited utility as predictive or diagnostic biomarkers because of the low positive predictive values [39]. In the liver damage induced by AC, the liver enzyme elevation pattern is commonly cholestatic, characterized by notable increases in alkaline phosphatase and gamma-glutamyl transpeptidase. However, in some cases, aminotransferase levels are significantly elevated, resulting in a mixed or hepatocellular pattern as well. These diverse phenotypes are also evidenced by the metabolomics analysis in our cohort study where we found patients showing pure cholestatic, hepatocellular or mixed phenotypes. The variability found among these DILI patients may also arise from the combined administration of two potential causal drugs (amoxicillin and clavulanic acid) with different toxicity mechanisms.

The immunosuppressive methotrexate drug is recognized for causing increases in serum aminotransferase levels, but prolonged therapy has also been associated with the development of fatty liver disease, cholestasis, fibrosis, and even cirrhosis [40,41]. Accordingly, the metabolomics data analysis from our DILI cohort evidenced dual hepatocellular and cholestatic phenotypes in patients.

Taken together, all of these results demonstrated the ability of metabolomics analysis to evidence the occurrence of variable DILI responses for certain drugs, while in others, the phenotype observed in patients is more homogeneous. The accurate and numerical classification of DILI episodes according to metabolomic data, revealed additional information to the categorization of DILI based on ALT and ALP levels and the R score classification, thus demonstrating that the metabolome analysis of patients can provide a faithful description of the metabolic status and alterations occurring in DILI patients.

Despite the potential relevance and clinical translatability of this study, we are aware of several limitations that, in our opinion, do not invalidate this proof-of-concept exercise. The work relies on a cohort of 79 well documented DILI patients, which comprises 31 different drugs. For some drugs, we had a relatively large number of cases to compare and to assess variability, while for other drugs, the number of DILI cases was quite limited. This might have biased the drug classification regarding the occurrence or not of variability in DILI responses of patients. Further endeavors are still needed to strengthen the clinical significance of the presented model. This would involve the utilization of complementary methods such as lipidomics and quantitative targeted metabolomics approaches, as well as ensuring the model validation is updated in additional DILI cohorts in forthcoming clinical studies.

To our knowledge, this study reports, for the first time, the use of metabolomics to reveal clear differences in the presentation and evolution of DILI in patients for whom the same drug was identified as the causative agent responsible for the DILI event. In addition, the results obtained indicate the presence of subtle metabolic alterations still linked to DILI in patients clinically classified as recovered. This information might be relevant for better patient follow up at late stages of the disease, as well as for anticipating a potential perpetuation of damage through other mechanisms (i.e., drug-induced autoimmune hepatitis). Another significant outcome of this research is that the predictive model developed allowed us to monitor variations in the class of sub-phenotype during disease progression. Thus, monitoring the metabolome might be an additional informative and helpful procedure to assess the DILI events in clinical practice.

## 4. Materials and Methods

### 4.1. Compliance with Ethical Standards

The present study was approved by the Ethics Committee for Biomedical Research of the Instituto de Investigación Sanitaria, Hospital Universitario y Politécnico La Fe (Valencia, Spain) (approval Nr. 2012/0452) and was conducted in accordance with the relevant guidelines, good clinical practices, and legal and ethical regulations. All patients gave written informed consent prior to participating in the clinical study.

### 4.2. Clinical Study: Patients’ Recruitment

In a study conducted between 2013 and 2018, 79 participating patients after providing written informed consent were referred to the Clinical Hepatotoxicity Unit for further DILI evaluation. The diagnosis of DILI followed international causality criteria as the well-established diagnostic scale RUCAM, considering factors such as clinical history, standard analytical results, chronological relationship, exclusion of alternative causes, use of hepatotoxic drugs, and high scores on causality scales (RUCAM > 6) [9,10]. Only episodes classified as highly probable (score 6 or higher) were included in the study.

Experienced clinicians established the diagnosis and classified hepatic damage as hepatocellular, cholestatic, or mixed-type DILI, according to R score. Patients were classified as cholestatic DILI if their ALP levels were ≥147 unit/L and had an R-score < 2; hepatocellular DILI if ALT levels were ≥56 unit/L and had an R-score ≥ 5, mixed DILI if the R-score fell between 2 and 5, being ALT ≥ 56 unit/L and ALP ≥ 147 unit/L, and finally recovered if ALT < 56 unit/L and ALP < 147 unit/L, and no clinical or analytical signs of disease were present. Blood samples were collected during scheduled clinical monitoring visits, and the number of samples varied depending on the follow-up and the duration of the recovery of each patient. A total of 278 plasma samples were collected and subjected to metabolomic analysis. These included 34 samples from patients with pure hepatocellular DILI, 79 samples from cholestatic DILI patients, 54 samples from mixed DILI patients, and 111 samples from patients who had clinically recovered. Patient data, such as gender, age, standard liver function indicators (ALT, gamma-glutamyl transferase (GGT), ALP, total bilirubin, and albumin) and other variables reflecting liver function, were also recorded alongside the collection of samples.

### 4.3. Standards and Reagents

Liquid chromatography–mass spectrometry (LC–MS) grade acetonitrile (CH_3_CN) and methanol (CH_3_OH) were obtained from Scharlau (Barcelona, Spain), and formic acid (HCOOH, ≥95%) from Sigma-Aldrich Química SL (Madrid, Spain). Ultra-pure water was generated employing a Milli-Q Integral Water Purification System from Merck Millipore (Darmstadt, Germany). Internal standards phenylalanine-D_5_, tryptophan-D_5_, and caffeine-D_9_ were purchased from C/D/N Isotopes Inc. (Quebec, QC, Canada).

### 4.4. Sample Preparation

**Figure 6:**
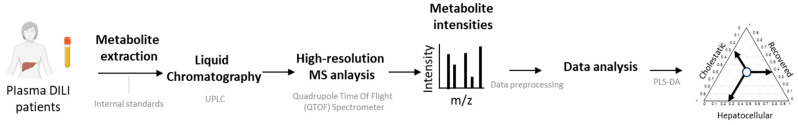
Experimental workflow of metabolomics analysis in DILI samples. Plasma samples were collected from DILI patients. Following metabolite extraction, metabolomics analysis was performed on UPLC-MS platform. Data obtained were subsequently preprocessed (peak table generation, batch effect correction, blank filtering, etc.) before PLS-DA models were generated. PLS-DA predicted values were integrated into a ternary plot to facilitate a visual and straightforward interpretation of a classification outcome (see Figure 1).

A flowchart of the methodology applied is shown in Figure 6. A 100 µL sample of the plasma fraction was thawed at room temperature. Subsequently, 300 µL of cold (4 °C) CH_3_OH was added for protein precipitation. The sample was homogenized using a vortex shaker for 10 s and centrifuged at 15,000× *g* for 10 min at 4 °C. Next, 300 µL of the supernatant was collected, and the solvent was evaporated under vacuum at 25 °C. The resulting residue was reconstituted in 150 µL of a 1 µM internal standard solution containing phenylalanine-D_5_, tryptophan-D_5_, and caffeine-D_9_ in H_2_0:CH_3_CN (98:2, 0.1% *v*/*v* HCOOH).

### 4.5. Metabolomic Analysis

Metabolomics analysis was performed using an Agilent 1290 Infinity UPLC system (Agilent Technologies, Santa Clara, CA, USA) with a Kinetex C18 column (Phenomenex, Torrance, CA, USA). The temperature of the autosampler and column was maintained at 4 °C and 55 °C, respectively, and the injection volume was 4 µL. A gradient elution method was employed at a flow rate of 400 µL/min, starting with 98% mobile phase A (H_2_0, 0.1% *v*/*v* HCOOH) for 0.5 min, followed by a linear gradient from 2 to 20% mobile phase A (H20, 0.1% *v*/*v* HCOOH) for 0.5 min, followed by a linear gradient from 2 to 20% mobile phase B (CH_3_CN, 0.1% *v*/*v* HCOOH) over 4 min and from 20 to 95% B over 4 min. After holding at 95% B for 1 min, a 0.25 min gradient was used to return to the initial conditions, which were maintained for 2.8 min. The mass spectrometry analysis was performed on an iFunnel QTOF Agilent 6550 spectrometer (Agilent Technologies, Santa Clara, CA, USA) in full scan mode, covering the *m*/*z* range of 70 to 1200. To ensure data accuracy, MS spectra recalibration was carried out by introducing reference standards into the source. For metabolite annotation, MS/MS data acquisition was performed with a collision energy of 25 V, and precursor ions were automatically selected in cycles. A specific *m*/*z* inclusion range was employed for repeated analysis of the QC samples.

### 4.6. Peak Table Generation and Batch Effect Correction

Each batch (ESI+/−) was processed individually using the XCMS software 2.7 in R [42], performing peak detection, integration, deconvolution, and alignment. The centWave method with specific parameters was employed for peak detection, and overlapping peaks were distinguished based on a minimum *m*/*z* difference. Intensity-weighted *m*/*z* values were calculated for each feature, and peak integration limits were determined using filtered data. Matching peaks across samples were achieved through the nearest method considering mz-retention time balance. To address missing data, raw data files were reintegrated in the regions of missing peaks. The accuracy of peak integration and alignment was evaluated by comparing automated and manual integration results.

Within-batch effect correction was carried out using the non-parametric QC-SVRC approach with a Radial Basis Function kernel [43]. Metabolite annotation was performed using MS/MS data and databases like the Human Metabolome Database and METLIN. The analysis included 278 samples and 828 annotated LC-MS features.

### 4.7. Software and Analysis

The *t*-test was utilized to evaluate the null hypothesis, examining whether the data from two groups (such as cholestatic DILI versus recovered patients) originated from independent random samples sharing equal means but with unknown and unequal variances. LC-MS features demonstrating *t*-test FDR-adjusted *p* values < 0.05 were deemed significantly altered and were selected accordingly.

For multivariate supervised analysis, PLS-DA was performed. Double cross-validation (2CV) with subject-wise cross-validation was used to estimate the out-of-sample prediction error of PLS-DA. Model development excluded patients classified as mixed-type and samples used for the development of the PLS models were excluded from the test sets. Cross-validation (CV) folds included all samples collected from each patient, and the selection of the model complexity (i.e., the number of latent variables, LVs) for each PLS model was based on the classification cross-validation accuracy, including 4 (cholestatic model), 5 (hepatocellular model), and 3 LVs (recovered model). For each PLS model, a VIP score threshold of 1 was used to select the most relevant features that were subsequently used to build the optimized PLS models including 291 (cholestatic model), 286 (hepatocellular model), and 298 features (recovered model). MATLAB R2021a (Mathworks Inc., Natick, MA, USA) and the PLS toolbox 9.0 (Eigenvector Research Inc., Wenatchee, WA, USA) were utilized for PLS-DA using in-house-written scripts. Raw data conversion for metabolite annotation was carried out using ProteoWizard. LipiDex software v1.1 [44] was employed for metabolite annotation, matching measured MS/MS spectra to an in silico library called LipidBlast.

## 5. Conclusions

Our research aimed to understand the diverse responses observed and to demonstrate the utility of metabolomics in characterizing and diagnosing different DILI phenotypes accurately. By identifying various metabolites in plasma and integrating this data into predictive models, we could assess the hepatocellular or cholestatic phenotype’s severity and assign numerical values to each specific DILI sub-phenotype’s contribution to the patient’s overall condition. This approach also allowed us to identify individual variations among DILI patients affected by the same drug. While some compounds exhibited consistent responses across patients, others displayed significant variability. Additionally, our analysis unveiled the potential transition from one sub-phenotype to another during disease progression, highlighting a spectrum of residual DILI metabolic features often overlooked in current clinical diagnosis and patient follow-up protocols.

## Data Availability

The peak tables and the dataset generated during the current study is available on the Zenodo repository under https://doi.org/10.5281/zenodo.10361027 accessed on 30 December 2023.

## Supplementary Materials

The following supporting information can be downloaded at: https://www.mdpi.com/article/10.3390/ijms25053003/s1.

## References

[1] Navarro V.J., Senior J.R. (2006). Drug-related hepatotoxicity. N. Engl. J. Med..

[2] Andrade R.J., Lucena M.I., Kaplowitz N., García-Muņoz B., Borraz Y., Pachkoria K., García-Cortés M., Fernández M.C., Pelaez G., Rodrigo L. (2006). Outcome of acute idiosyncratic drug-induced liver injury: Long-term follow-up in a hepatotoxicity registry. Hepatology.

[3] Andrade R.J., Lucena M.I., Fernández M.C., Pelaez G., Pachkoria K., García-Ruiz E., García-Muñoz B., González-Grande R., Pizarro A., Durán J.A. (2005). Drug-induced liver injury: An analysis of 461 incidences submitted to the Spanish registry over a 10-year period. Gastroenterology.

[4] Björnsson E., Davidsdottir L. (2009). The long-term follow-up after idiosyncratic drug-induced liver injury with jaundice. J. Hepatol..

[5] Björnsson E.S., Björnsson H.K. (2017). Mortality associated with drug-induced liver injury (DILI). Transl. Gastroenterol. Hepatol..

[6] Teschke R., Uetrecht J. (2021). Mechanism of idiosyncratic drug induced liver injury (DILI): Unresolved basic issues. Ann. Transl. Med..

[7] Hosack T., Damry D., Biswas S. (2023). Drug-induced liver injury: A comprehensive review. Ther. Adv. Gastroenterol..

[8] Vega M., Verma M., Beswick D., Bey S., Hossack J., Merriman N., Shah A., Navarro V., Drug Induced Liver Injury Network (2017). The incidence of drug- and herbal and dietary supplement-induced liver injury: Preliminary findings from gastroenterologist-based surveillance in the population of the State of Delaware. Drug Saf..

[9] Jee A., Sernoskie S.C., Uetrecht J. (2021). Idiosyncratic Drug-Induced Liver Injury: Mechanistic and Clinical Challenges. Int. J. Mol. Sci..

[10] Benichou C., Danan G., Flahault A. (1993). Causality assessment of adverse reactions to drugs—II. An original model for validation of drug causality assessment methods: Case reports with positive rechallenge. J. Clin. Epidemiol..

[11] Danan G., Teschke R. (2016). RUCAM in Drug and Herb Induced Liver Injury: The Update. Int. J. Mol. Sci..

[12] Lu R.-J., Zhang Y., Tang F.-L., Zheng Z.-W., Fan Z.-D., Zhu S.-M., Qian X.-F., Liu N.-N. (2016). Clinical characteristics of drug-induced liver injury and related risk factors. Exp. Ther. Med..

[13] Martínez-Sena T., Moro E., Moreno-Torres M., Quintás G., Hengstler J., Castell J.V. (2023). Metabolomics-based strategy to assess drug hepatotoxicity and uncover the mechanisms of hepatotoxicity involved. Arch. Toxicol..

[14] Moreno-Torres M., Quintás G., Castell J.V. (2022). The Potential Role of Metabolomics in Drug-Induced Liver Injury (DILI) Assessment. Metabolites.

[15] Quintás G., Castell J.V., Moreno-Torres M. (2023). The assessment of the potential hepatotoxicity of new drugs by in vitro metabolomics. Front. Pharmacol..

[16] Quintás G., Martínez-Sena T., Conde I., Pareja Ibars E., Kleinjans J., Castell J.V. (2021). Metabolomic analysis to discriminate drug-induced liver injury (DILI) phenotypes. Arch. Toxicol..

[17] Petrovic A., Vukadin S., Sikora R., Bojanic K., Smolic R., Plavec D., Wu G.Y., Smolic M. (2022). Anabolic androgenic steroid-induced liver injury: An update. World J. Gastroenterol..

[18] Debureaux P.-E., Nailly D.L.F.D., Tavernier E., Bedoui M., Kuhnowski F., Tamburini J., Fornecker L.-M., Camus V., Sibon D., Moles M.-P. (2020). Sinusoidal obstruction syndrome: A warning about autologous stem cell transplantation preceded by regimens containing oxaliplatin. Bone Marrow Transplant..

[19] Morris-Stiff G., Tan Y.M., Vauthey J.N. (2008). Hepatic complications following preoperative chemotherapy with oxaliplatin or irinotecan for hepatic colorectal metastases. Eur. J. Surg. Oncol..

[20] Mohammadi O., Kassim T.A. (2023). Azathioprine. StatPearls.

[21] Moses V., Ramakrishna B., Thomas K. (2011). Azathioprine induced cholestatic hepatitis. Indian J. Pharmacol..

[22] Ramachandran A., Jaeschke H. (2019). Acetaminophen Hepatotoxicity. Semin. Liver Dis..

[23] Goyal L., Madabhushi A.K., Siddiqi M.S., Kale S., Mallick D.C. (2022). Severe Case of Cholestatic Hepatitis from Amoxicillin/Clavulanic Acid. Cureus.

[24] deLemos A.S., Ghabril M., Rockey D.C., Gu J., Barnhart H.X., Fontana R.J., Kleiner D.E., Bonkovsky H.L., Drug-Induced Liver Injury Network (DILIN) (2016). Amoxicillin–Clavulanate-Induced Liver Injury. Dig. Dis. Sci..

[25] Beraldo D.O., Melo J.F., Bonfim A.V., Teixeira A.A., Teixeira R.A., Duarte A.L. (2013). Acute cholestatic hepatitis caused by amoxicillin/clavulanate. World J. Gastroenterol..

[26] West S.G. (1997). Methotrexate hepatotoxicity. Rheum. Dis. Clin. N. Am..

[27] García-Cortés M., Stephens C., Lucena M.I., Fernández-Castañer A., Andrade R.J. (2011). Causality assessment methods in drug induced liver injury: Strengths and weaknesses. J. Hepatol..

[28] Russmann S., Kullak-Ublick G.A., Grattagliano I. (2009). Current concepts of mechanisms in drug-induced hepatotoxicity. Curr. Med. Chem..

[29] Tiller N.B., Stringer W.W. (2023). Exercise-induced increases in “liver function tests” in a healthy adult male: Is there a knowledge gap in primary care?. J. Fam. Med. Prim. Care.

[30] Ewid M., Sherif H., Allihimy A.S., Alharbi S.A., Aldrewesh D.A., Alkuraydis S.A., Abazid R. (2020). AST/ALT ratio predicts the functional severity of chronic heart failure with reduced left ventricular ejection fraction. BMC Res. Notes.

[31] Watkins P.B., Kaplowitz N., DeLeve L.D. (2013). Chapter 17—Biomarkers for Drug-Induced Liver Injury. Drug-Induced Liver Disease.

[32] Devarbhavi H. (2012). An Update on Drug-induced Liver Injury. J. Clin. Exp. Hepatol..

[33] Aithal G., Watkins P., Andrade R., Larrey D., Molokhia M., Takikawa H., Hunt C.M., A Wilke R., Avigan M., Kaplowitz N. (2011). Case Definition and Phenotype Standardization in Drug-Induced Liver Injury. Clin. Pharmacol. Ther..

[34] Zhu C., Ren X., Liu D., Zhang C. (2021). Oxaliplatin-induced hepatic sinusoidal obstruction syndrome. Toxicology.

[35] Liu F., Cao X., Ye J., Pan X., Kan X., Song Y. (2018). Oxaliplatin-induced hepatic sinusoidal obstruction syndrome in a patient with gastric cancer: A case report. Mol. Clin. Oncol..

[36] Xie M., Hajifathalian K., Lucero C., Fulmer C.G., Yantiss R.K. (2018). Azathioprine-Induced Cholestatic Liver Injury with Mild Ductopenia: 1386. Off. J. Am. Coll. Gastroenterol..

[37] Lindgren A., Aldenborg F., Norkrans G., Olaison L., Olsson R. (1997). Paracetamol-induced cholestatic and granulomatous liver injuries. J. Intern. Med..

[38] Yoon E., Babar A., Choudhary M., Kutner M., Pyrsopoulos N. (2016). Acetaminophen-Induced Hepatotoxicity: A Comprehensive Update. J. Clin. Transl. Hepatol..

[39] Lucena M.I., Molokhia M., Shen Y., Urban T.J., Aithal G.P., Andrade R.J., Day C.P., Ruiz–Cabello F., Donaldson P.T., Stephens C. (2011). Susceptibility to amoxicillin-clavulanate-induced liver injury is influenced by multiple HLA class I and II alleles. Gastroenterology.

[40] Atayan Y., Cagın Y.F., Erdogan M.A., Bilgic Y., Bestas R., Aladag M. (2015). Prolonged Cholestatic Liver Disease Secondary to Methotrexate. Med. Sci.|Int. Med. J..

[41] Ezhilarasan D. (2021). Hepatotoxic potentials of methotrexate: Understanding the possible toxicological molecular mechanisms. Toxicology.

[42] Smith C.A., Want E.J., O’Maille G., Abagyan R., Siuzdak G. (2006). XCMS:  Processing Mass Spectrometry Data for Metabolite Profiling Using Nonlinear Peak Alignment, Matching, and Identification|Analytical Chemistry. Anal. Chem..

[43] Kuligowski J., Sánchez-Illana Á., Sanjuán-Herráez D., Vento M., Quintás G. (2015). Intra-batch effect correction in liquid chromatography-mass spectrometry using quality control samples and support vector regression (QC-SVRC). Analyst.

[44] Hutchins P.D., Russell J.D., Coon J.J. (2018). LipiDex: An Integrated Software Package for High-Confidence Lipid Identification. Cell Syst..

